# Risk Factors of Rejection in Renal Transplant Recipients: A Narrative Review

**DOI:** 10.3390/jcm11051392

**Published:** 2022-03-03

**Authors:** Hani Oweira, Ali Ramouz, Omid Ghamarnejad, Elias Khajeh, Sadeq Ali-Hasan-Al-Saegh, Rajan Nikbakhsh, Christoph Reißfelder, Nuh Rahbari, Arianeb Mehrabi, Mahmoud Sadeghi

**Affiliations:** 1 Department of Surgery, Medical Faculty Mannheim, University of Heidelberg, 68167 Mannheim, Germany; hani.oweira@umm.de (H.O.); christoph.reissenfelder@umm.de (C.R.); nuh.rahbari@umm.de (N.R.); 2 Department of General, Visceral, and Transplantation Surgery, University of Heidelberg, 69120 Heidelberg, Germany; ali.ramouz@med.uni-heidelberg.de (A.R.); omid.ghamarnejad@med.uni-heidelberg.de (O.G.); elias.khajeh@med.uni-heidelberg.de (E.K.); sadeq.ali-hasan-al-saegh@med.uni-heidelberg.de (S.A.-H.-A.-S.); rajan.nikbakhsh@med.uni-heidelberg.de (R.N.); 3 Department of Transplantation Immunology, University of Heidelberg, 69120 Heidelberg, Germany; mahmoud.sadeghi@med.uni-heidelberg.de

**Keywords:** kidney transplantation, rejection, recipient

## Abstract

Multiple factors influence graft rejection after kidney transplantation. Pre-operative factors affecting graft function and survival include donor and recipient characteristics such as age, gender, race, and immunologic compatibility. In addition, several peri- and post-operative parameters affect graft function and rejection, such as cold and warm ischemia times, and post-operative immunosuppressive treatment. Exposure to non-self-human leucocyte antigens (HLAs) prior to transplantation up-regulates the recipient’s immune system. A higher rate of acute rejection is observed in transplant recipients with a history of pregnancies or significant exposure to blood products because these patients have higher panel reactive antibody (PRA) levels. Identifying these risk factors will help physicians to reduce the risk of allograft rejection, thereby promoting graft survival. In the current review, we summarize the existing literature on donor- and recipient-related risk factors of graft rejection and graft loss following kidney transplantation.

## 1. Introduction

Kidney transplantation (KTx) is the treatment of choice for end-stage renal diseases (ESRD) [1]. KTx improves the patients’ quality of life and life expectancy compared with other renal replacement therapies such as dialysis. Furthermore, advances in immunosuppressive therapies have substantially improved KTx outcomes [2]. Although long-term graft outcomes have improved noticeably through recent decades, survival of KTx recipients is fourfold lower than in individuals without ESRD. Graft rejection is one of the main causes of graft loss after KTx, so understanding the factors affecting graft rejection is important for promoting graft survival.

Multiple factors influence graft rejection after KTx [1,3]. Pre-operative factors affecting graft function and survival include donor and recipient characteristics such as age, gender, race, and immunologic compatibility [4,5]. In addition, several peri- and post-operative parameters affect graft function and rejection, such as cold and warm ischemia times, and post-operative immunosuppressive treatment [6,7,8]. Identifying these risk factors will help physicians to reduce the risk of allograft rejection, thereby promoting graft survival.

In the current review, we summarize the existing literature on donor- and recipient-related risk factors of graft rejection and graft loss following KTx.

## 2. Donor-Related Factors

### 2.1. Donor Gender

Using the large Collaborative Transplant Study database, Zeier et al. showed that death-censored graft survival and five-year graft survival were significantly lower in patients receiving grafts from female donors [9]. The rate of graft loss among patients receiving organs from female donors was noticeably higher during the first five years after KTx [10]. However, a retrospective survival analysis of 766 KTx patients showed comparable graft survival rates between organs from male and female donors [11]. In terms of short-term outcomes, some studies have shown that grafts from female donors have fewer nephrons and are more susceptible to immunosuppressive-induced nephrotoxicity than grafts from male donors [12]. However, the protective and stimulatory effects of female hormones, such as estradiol, improve graft function and reduce cellular infiltration, thereby providing better long-term outcomes [12]. These findings are supported by a prospective study, which suggested a higher risk of acute rejection when grafts were transplanted from a female donor, and a higher risk of complete graft loss after five years when grafts were transplanted from a male donor.

Gender compatibility between donors and recipients may influence KTx outcomes, but there is no consensus on donor-recipient gender matching in KTx. Some studies have shown that transplanting from female donors into male recipients increases the risk of early rejection, and that transplanting from male donors into female recipients increases the risk of early graft loss compared with all other gender combinations, suggesting that gender matching may improve KTx outcome.

### 2.2. Donor Age

Donor age is a better predictor of KTx outcome than donor gender. Allografts from older donors have a higher risk of post-transplant complications, delayed graft function, acute rejection, and graft failure [13]. Transplantation from both very young and very old donors is a risk factor for poor transplant outcome [9]. The risk ratio was higher when kidneys were donated by younger female donors (16 to 45 years) than by older female donors (>45 years) and then transplanted into male recipients [14]. Recent studies have suggested grafts can be collected from donors older than 50 years as graft survival rates are comparable with those from younger donors. However, grafts from donors older than 65 years have a higher rate of acute rejection. Although recent studies have shown that old to young or young to old KTx does not increase allograft rejection, transplant from old donors could reduce generally the long-term allograft survival, and thereby should be transplanted in older recipients [15].

### 2.3. Living versus Deceased Donor

Organs procured from living donors provide several benefits by reducing warm and cold ischemia times and the immunological characteristics can be precisely evaluated before transplantation [14]. Living donor grafts reduced the rate of short-term morbidity and mortality, and increased graft survival. Living donors with diabetes mellitus and hypertension are disqualified from donating organs because of the increased risk for ESRD [16]. Immunological activation is also lower in living donors; 53% of deceased-donor renal allografts had increased neutrophil infiltration compared with 0% of living-donor grafts. However, there are still reasons to consider deceased-donor transplantation. The risk of mortality is 68% lower in deceased-donor kidney recipients than in similar patients who do not receive a transplant [14]. The lack of equilibrium between organ supply and clinical demand still prohibits us from obtaining the ideal transplant scenario in every case, despite advances in organ transplantation. Therefore, we must continue to procure organs from deceased donors, despite the associated risks, to increase the donor pool and meet the demand for organs [17,18].

### 2.4. Non-Marginal and Marginal Donors

Extended donor criteria were defined by Port et al. in 2002. However, the marginal kidney donor criteria remain unclear, despite progressive expansion of transplant waiting lists [19]. Physicians mostly might not follow the defined or their own center’s criteria for organ procurement, particularly regarding circulatory death of the donor. In the literature, different criteria have been suggested for definition of marginal kidney donors. Kidney donors with brain dead were considered marginal, if were aged >60 years, or >50 years with any of the following conditions: (1) hypertension, (2) cerebro-vascular cause of brain death or (3) pre-retrieval serum creatinine (SCr) level > 1.5 mg/dL, with a degree of glomerulosclerosis >15% and prolonged cold ischemia. Additionally, marginal living donors are considered to be older (>70 years old), obese (>35 kg/m^2^), and have hypertension, diabetes, nephropathies, and kidney cysts [20]. The one-year graft function was comparable between organs obtained from marginal and non-marginal donors, but rates of interstitial fibrosis/tubular atrophy and acute rejection were higher in organs from marginal donors. Dual-kidney transplant from marginal donors into a single recipient increases the available organs and prevents disqualification of marginal organs. Recent studies have shown a diminished risk of adverse effects after KTx from marginal donors when the CIT was short [21]. Pre-transplant biopsies can be taken to evaluate organ quality (20). Other techniques have also been used to increase the quality of donor organs, even from marginal donors, such as normothermic ex vivo and post-mortem perfusion [22,23].

## 3. Recipient-Dependent Factors

### 3.1. Recipient Race

African American populations are at a higher risk of suboptimal renal transplantation [24]. According to several trials and large transplant cohorts, African American patients have higher rates of acute rejection and early graft failure compared with Caucasians [24]. Out of 15,000 KTxs performed in 2002, only 22.5% of recipients were Black and 13% were Hispanic. Hispanic ethnicity was identified as an independent indicator of premature graft failure.

### 3.2. Recipient Age

A younger age of recipients is associated with an elevated risk of suboptimal allograft outcomes. The absorption, delivery, and excretion of immunosuppressive drugs is different in pediatric and adult patients, and pediatric patients have a higher relative risk of acute rejection [25,26]. Current immunosuppression and standardized induction therapy have reduced the probability of acute graft rejection in pediatric patients and have increased the long-term allograft survival [26,27]. The recipients aged 6–12 years had a lower risk of graft loss within 90 days of transplantation than recipients aged 2–5 and 13–20 years. The most common reason for early allograft failure was functional complications (thrombosis and primary non-function) in patients younger than five years and non-compliance-induced acute rejection in juveniles [27,28]. Older patients are more likely to need a KTx as the chance of developing ESRD rises dramatically after 60 years.

Another important issue, which is raised to evaluate the role of recipient age on outcomes of kidney transplantation, is the adherence of the patients to post-transplant therapies. Non-adherence has serious consequences for kidney transplant recipients, including higher healthcare costs, medical problems, allograft rejection and loss, and patient mortality. A recent systematic review of the literature reported discouraging outcomes in pediatric patients, concerning the adherence after organ transplantation. Non-adherent pediatric patients, after organ transplantation are at a twofold higher risk of biopsy-proven acute rejection and hospitalization, which lead to organ loss in 80% of these patients. Estimates of non-adherence among pediatric renal transplant recipients range from 30 to 70%. On the other hand, it has been demonstrated that each 10% decrement in adherence is associated with an 8% higher hazard of graft failure and mortality [29]. Despite the considerable burden of non-adherence after kidney transplantation, no systematic approach has been introduced to deal with it and further studies are needed to identify the influencing factors and their management.

### 3.3. Concomitant Diseases

Concomitant diseases, such as infectious disease, coagulopathies, and malignancies can affect post-KTx outcomes [30]. The leading cause of death after KTx is cardiovascular disease, particularly in patients suffering from autoimmune diseases such as lupus erythematous. Antiphospholipid antibodies and cardiovascular disease should be evaluated before KTx, with due attention to the increased risk of intravascular thrombosis and the recurrence of lupus nephritis.

### 3.4. Re-Transplantation

Acute rejection rates are high (from 33% to 69%) among renal re-transplant patients [31], and there are growing numbers of patients awaiting re-transplantation. Managing these patients is challenging because the rate of hyperimmunity increases with positive crossmatch KTx [31]. In addition, HLA mismatch could lead to increase rate of acute rejection among this group of patients [32]. Previously, presence of repeated HLA antigen mismatched from the first transplant was associated with rapid and early alloimmune damage as well as graft loss, by rechallenging the immune system of recipient. However, recent studies have showed that patients with repeated HLA antigen mismatched class II, who are sensitized or nephrectomized have higher tendency to develop rejection and graft loss after re-transplantation. Although re-transplantation recipients are at risk of delayed graft function due to early acute rejection (prior sensitization), re-transplantation still offers considerable benefits [33,34].

## 4. Donor-Recipient Compatibility

### 4.1. ABO Blood Types

The ABO blood typing system is based on a group of antigens located on the erythrocyte surface. These antigens induce antibody development upon exposure to a foreign host immune system. Incompatibility with the main blood group antigens (A, B, AB, and A1) is clinically important because of naturally circulating immunoglobulin (Ig)M and IgG antibodies. Transplantation between individuals who are not blood-group-compatible results in hyperacute rejection, and this incompatibility has traditionally been considered a contraindication to transplantation. Because of the organ shortage, pre-transplant splenectomy, plasmapheresis, and immunosuppression have been performed to overcome compatibility issues [35]. In 2017, Masutani et al. reported comparable results of histological rejection and infectious complications between ABO-compatible KTx and ABO-incompatible KTx under desensitization protocol of low-dose Rituximab and plasmapheresis with maintenance and induction of immunosuppression [36]. The importance of selecting organs from donors with A2 blood type for recipients with other blood types such as O and B has been investigated. Twenty percent of patients with blood group A have type A2 blood, which reduces the immunological risk of graft loss. A-group antigens are negligible in individuals with type A2 blood, much like the universal donor blood type O. Nonetheless, serum anti-A1 antibody levels should still be assessed in these individuals. However, several disadvantages have been demonstrated for this approach, including high costs, higher risk of rejections, infections, and poor organ survivals.

On the other hand, paired donor exchange is a novel program that seems to provide promising outcomes, concerning the primary outcomes, especially from US. In this program, kidneys are exchanged between two or more HLA-incompatible or ABO-incompatible living donor and recipients in order to achieve better compatibility of the organs. However, ethical considerations have restricted these approaches in some countries, such as Japan. Therefore, ABO-incompatible kidney transplantation (ABOi) has been implemented in Japan, and has shown acceptable outcomes for patients with ESKD. ABOi may increase the frequency of kidney transplants and may lead to the shortening of dialysis therapy or even its avoidance [37]. Morath et al. also reported that ABOi kidney transplant increased the donor pool up to 30%. However, it should not be ignored that ABO-compatible transplantation is still first-choice, since ABOi is correlated with higher risk of early rejection, infection, and subsequent mortality.

### 4.2. Human Leucocyte Antigen Typing

Exposure to non-self HLAs increases the risk of graft rejection and early graft failure. Pre-transplantation sensitivity to HLA antigens in the recipient stimulates clonal proliferation of lymphocytes and antibodies to donor tissue [38]. Graft survival in patients with peak or current panel reactive antibody (PRA) level ≥ 50% is half that of patients with a PRA level <50% [39,40]. This consequence is exacerbated in re-transplant recipients whose survival of the graft declines by an estimated rate of 10% [41]. Transplant patients with a history of abortions or extensive exposure to blood products have a greater risk of acute rejection, which might be associated with peak or elevated PRA levels [41]. However, the current standardization of single antigen has diminished the popularity of the PRA. It has been shown that a complement-dependent lymphocytotoxic crossmatch (CDC-XM) test can predict the possible immunological risks in KTx. Therefore, this method has been established as the gold standard test for graft allocation and could be utilized before renal transplantation. Nevertheless, this test could not detect specifically the preexisting donor-specific HLA antibodies (HLA-DSA). In this regard, new methods with solid-phase assays helped us to detect HLA antibodies more sensitive and specific [42]. As a new method, the analysis of serum with the beads covered by a single HLA antigen (single antigen bead-SAB) has been developed to improve the sensitivity of HLA antibody detection to prevent graft rejection [43]. As mentioned above, sensitization of recipient to donor HLA plays an important role in the prognosis of renal transplantation. This Sensitization could be demonstrated by the amount of donor-specific anti-HLA IgG antibodies (DSA) in recipient sera, which could be measured via the cell-based complement-dependent cytotoxicity crossmatch (CDC-XM) assay. In addition to CDC-XM, the assessment of the complement-binding capacity of donor-specific anti-HLA antibodies shows promising results to detect patients, who are categorized at high risk level for KTx [44]. Flow cytometry-based solid phase assays (flow-beads) offer a sensitivity for detecting particular HLA that is at least comparable to flow cytometer crossmatch (FCXM) [45]. As a result, HLA-DSAs can be detected without an FCXM test by comparing the recipient’s HLA antibody specificities to the donor’s HLA-typing (i.e., virtual XM). Virtual XM can determine whether DSA is present or not, and it could become a valuable tool for determining organ allocation and pretransplant risk level [46].

HLA tissue typing and measurement of anti-donor HLA antibody serum levels in the recipients is carried out before transplantation. HLA tissue typing determines the allocation of deceased-donor organs. HLA class I molecules (HLA-A, -B, or -C) are found on nearly all nucleated cells in the body, and lymphocytes from the lymph node, blood, or spleen can be used for tissue typing. HLA class II molecules (HLA-DR or -DQ) are present on lymphocytes (specifically lymphocytes B), APCs, and endothelial microvascular cells [39]. HLA typing identifies the six major histocompatibility complex (MHC) or HLA (-A, -B, and -DR) molecules of the donor and receiver [47]. These antigens are aligned between the donor and recipient to predict the transplant outcome. Thanks to a substantial improvement in survival of the graft, a six-antigen alignment or zero-antigen mismatch between a deceased donor and a prospective recipient allows preferential distribution of organs [48]. Furthermore, an HLA match outweighed the negative effect of a long CIT in KTx patients [41]. Six-antigen-matched kidneys have the best allograft survival, and many physicians consider all associated HLAs when evaluating the risk of acute rejection and overall allograft efficiency. The degree of HLA mismatch has been associated with allograft outcome. Opelz et al. evaluated the impact of HLA matching on graft survival in over 9000 deceased-donor transplants. HLA-A and -B matching was associated with an 8% increase in one-year lung transplantation survival rate, HLA-DR matching with a 10% increase, and HLA-B + DR matching with a 19% increase [40]. Opelz et al. have also shown that HLA compatibility significantly improves graft and patient survival in a study including more than 150,000 recipients [49]. During a 10-year post-transplantation follow-up, the survival rate of deceased-donor kidney transplants with a complete mismatch (6 HLA-A+B+DR mismatches) was 17% lower than that of non-mismatched grafts. It has also been estimated that the class II HLA-DR locus had a greater effect on graft outcome during the first post-transplant year than class I HLA-A and HLA-B loci. HLA class I antibodies have been associated with a higher risk of delayed graft function and acute rejection episodes within the first 3 months after transplantation. CD8+ T cell migration, NK cell restoration, and B lymphocyte restriction can describe the general immunological state after kidney transplantation. Furthermore, the amount of the regulatory T-cells (Treg) population early after transplantation and FOXP3 gene expression are linked to allograft function [50]. In addition, terminally differentiated effector memory (TEMRA) CD8+ T cells can implicate in humoral and cellular rejection and are considered as a good predictor marker to monitor the development of potential graft failure after KTx [51]. In another study, it has been shown that preformed T-cell alloreactivity and HLA eplet mismatch assessment may refine current baseline immune-risk stratification in KTx [52]. B cells play a key part in alloimmunity regulation, and they have been identified as a group of genes that could be used to screen outcomes of renal transplant recipients on a larger scale [53]. There are also some urine biomarkers which are correlated with allograft injury including CXCL9, CXCL10, C-C motif chemokine ligand 2 (CCL2), NGAL, IL-18, cystatin C, KIM1, T-cell immunoglobulin, and mucine domains-containing protein 3 (TIM3) [54]. Nonetheless, translating and validating the predictive role of these biomarkers in real-world scenarios and incorporating them into normal clinical practice, remains a challenge [55].

Kidney transplant success and graft survival were not predictable until the late 1960’s because the effects of a positive crossmatch and high PRA incidence were not fully understood. Evaluating the kidney recipient serums against a sample of unknown donor lymphocytes, showed an 80% decrease in organ survival among patients with a successful crossmatch, indicating the pre-existing antibodies to donor lymphocytes in the recipient serum. The rate of immediate graft loss was significantly higher in female patients, particularly in those with a history of pregnancy. These findings formed the basis for a novel immunological risk evaluation and patient stratification as ‘high immunological risk’. Donor-specific antibodies are identified to predict the immunological risk of rejection after transplantation. Determining the PRA reactivity is an important pre-transplantation assessment because it assesses the presence of antibodies against class I and II HLA antigens in the serum of the recipient that could be responsible for hyperacute or acute rejection [39]. In fact, the presence of modest levels of circulating antibodies has been associated with an increased risk of acute graft rejection and graft failure [39]. Opelz et al. stated that in HLA-identical sibling-kidney transplantations, PRAs against non-HLAs is closely correlated with long-term graft failure [56]. Comparing more than 4000 HLA-identical sibling transplantations with approximately 160,000 cadaveric grafts showed that PRAs in deceased-donor grafts reduced graft survival in the first year when compared with graft survival of HLA-identical grafts [56]. Long-term follow-up in the HLA-identical group revealed a big impact for PRAs in recipients with elevated PRA levels (>50%), whereas the number of functioning grafts after 10 years was slightly reduced [56]. Further studies redefined the importance of anti-HLA class I antibodies in acute rejection [47]. Acute rejection has been associated with anti-HLA class I antibodies in patients with negative crossmatch prior to transplantation. The risk of acute vasculitis and glomerulitis and fibrin thrombi and fibrinoid necrosis is higher in patients with anti-HLA class I antibodies.

Currently, clinical application of biomarkers has been developed to obtain a better therapeutic regimen with immunosuppressive agents [57], since these biomarkers might play a role in prediction of allograft rejection and could help with therapeutic decision-making [58]. Recently, it has been suggested that T follicular helper cells (Tfhs) could promote DSA appearance, and monitoring of activated Thfs early after transplantation may help to predict the DSA appearance after renal transplantation and choose a better therapeutic target with immunosuppressive agents [59].

## 5. Perioperative Factors

### Ischemia/Reperfusion Injury

Ischemia/reperfusion injury is one of the most common complications after renal transplantation. It is influenced by the warm ischemia time (WIT) and CIT. Adenosine triphosphate production is decreased in tubular cells because of oxygen deficiency after prolonged periods of ischemia, which alters enzyme activity. After reperfusion, free-oxygen radical production induces local inflammation and stimulates the complement and coagulation cascade. While preservation solutions and cold temperature maintain electrolyte balance by diminishing the rate of metabolism in the tubular cells, prolonged ischemia increases anaerobic respiration [48]. Ischemia/reperfusion injury delays graft activity, which is characterized by acute tubular necrosis.

Several studies have shown that prolonged CIT and WIT increases graft alloreactivity and acute graft rejection [60]. The CIT and WIT has the greatest impact on the survival of grafts from deceased donors and marginal donors, so reducing the ischemia time will improve the longevity and utility of these marginal donor kidneys [61]. Prolonged CIT increases the humoral antibody response [23]. Patients with identical demographics and baseline data were divided based on the CIT (less and more than 15 h). The outcomes showed that three or four HLA-A and -B mismatches and a CIT ≥ 15 h increased the risk of graft loss. Patients with a CIT ≥ 15 h produced more class I antibodies. Therefore, it can be hypothesized that a CIT of more than 15 h independently contributes to the higher production of class I AHG PRA, and subsequently higher graft rejection. Prolonged CIT (>30 h compared with <10 h) was associated with a monotonic increase in the relative risk of graft loss [62]. Because lymphocytes have been demonstrated to mediate transplant rejection, reduction of these cells has been investigated as a way to prevent rejection and possibly induce immunologic tolerance. Monoclonal antibodies, cytotoxic medicines, and radiation have all been shown to significantly decrease lymphocytes. The use of depletional agents as an induction therapy has also been growing and is used in 59% of adult kidney transplant recipients. In this regard, Lymphocyte depletion prior to or beginning at the time of transplantation is beneficial in reducing maintenance immunosuppression [63]. Machine perfusion has had a renewal in the last 10–15 years over static cold storage (SCS). Hypothermic machine perfusion (HMP) has been used as a machine perfusion to reduce the rates of delayed graft function (DGF) in comparison to SCS with significant improving of the overall graft survival. Although HMP attenuates the rates of DGF, its effect on long-term renal and patient outcomes is not yet clear. There is limited clinical literature in the use of normothermic machine perfusion (NMP), but a few pilot studies have shown its potential to resuscitate commonly discarded kidneys. In addition to preservation, machine perfusion also allows for various diagnostic and therapeutic interventions during the preservation period to assess and optimize the viability of the procured kidney [64].

## 6. Post-Transplant Factors

### 6.1. Delayed Graft Function

The frequency of delayed graft function varies from 4 to 10% in living donor transplants and from 5 to 50% in deceased-donor kidney transplants. Although the association between delayed graft function and rates of rejection has not been yet clearly described by the studies, it has been suggested that early detection of patients at risk of delayed graft function will allow early post-operative hemodynamic and immunosuppressive treatment to promote graft function [65]. T-cell-depletion (e.g., using calcineurin inhibitors) might improve perfusion and recovery of the graft by delaying nephrotoxic immunosuppression [65,66,67].

### 6.2. Immunosuppressive Regimen

New immunosuppressive regimens are accompanied with better monitoring and desensitization strategies have been utilized to extend the donor criteria [68,69]. Currently, immunosuppressive agents can be classified into three categories: “induction agents”, “maintenance therapy” and “treatment for rejection”. Induction agents are typically polyclonal antibodies (anti-thymocyte globulins) and interleukin (IL)-2 receptor antagonists (basiliximab). New induction agents include alemtuzumab, efalizumab and alefacept. The four drug classes that comprise maintenance regimens include calcineurin inhibitors (cyclosporine and tacrolimus), mTOR inhibitors (sirolimus and everolimus), antiproliferative agents (azathioprine and mycophenolic acid), and corticosteroid. Nowadays, the current standard of care for kidney transplant immunosuppression is a calcineurin inhibitor–based immunosuppressive regimen with tacrolimus and mycophenolate [70]. These agents are currently administered in approximately 90% of the patients, with or without adjuvant steroid therapy [71]. Three new maintenance agents with novel mechanisms of action include: sotrastaurin, a protein kinase C inhibitor; belatacept, a recently approved costimulation blocker; and tofacitinib, a JAK 3 inhibitor. However, in contrast to sotrastaurin and tofacitinib, belatacept has been used widely in clinical practice as it has immunosuppressive effects without showing renal and non-renal toxicities associated with calcineurin inhibitors. In a phase III study, belatacept demonstrated better renal function and similar graft/patient survival at 1-year post-transplant compared to cyclosporine, despite a higher rate of acute rejection in EBV negative patients (BENEFIT study) [72]. At 3-year post-transplantation, extended criteria donor (ECD) kidney transplant recipients under belatacept-based immunosuppression achieved better renal function, similar graft/patient survival, increased risk of posttransplant lymphoproliferative disorder (PTLD), and lower risk of cardiovascular/metabolic profile in comparison to other group pf patients treated with cyclosporine (BENEFIT-EXT study) [73].

Induction treatment by multiple agents such as thymoglobulin, IL-2 receptor antibody, and other antibodies can decrease the risk of cellular rejection in recipients of kidney transplantation [74,75]. A meta-analysis has showed that early steroid avoidance (defined as 14 days of steroid therapy) in patients receiving induction and tacrolimus-based maintenance therapy was found to be efficacious and safe in terms of graft, and patients who were randomized to early steroid avoidance received induction therapies [76]. It has been illustrated that Tacrolimus has a lower risk of rejection in multiple randomized clinical trials compared to Cyclosporin or mTOR inhibitors, as treatment with Tacrolimus is associated with a significantly better cardiovascular risk profile and superior renal function compared with cyclosporin microemulsion translate into improved long-term graft survival [77,78]. Recent trials demonstrate that tacrolimus has a superior 1-year graft survival rate than cyclosporine, however with a higher rate of post-transplant diabetes mellitus (PTDM). These findings, together with decreased pharmacokinetic variability and an arguably better side effect profile than cyclosporine, have led to an increase in tacrolimus use in clinical practice. Due to the small therapeutic window, significant fluctuations in tacrolimus trough levels early after transplantation may result in a poor clinical outcome [79]. Furthermore, low tacrolimus trough levels may not be beneficial in preventing acute rejection (AR), whereas high levels are linked to increased infection and toxicity [80]. Because of the low medication compliance of patients after transplantation for Tacrolimus, its once-daily dosage form has just been introduced and is currently being tested in clinical trials in order to improve the adherence of the medication [81]. The use of prolonged-release tacrolimus has shown to be effective in several investigations. The impending usage of this drug in the transplant population could attenuate non-compliance difficulties. In comparison to its higher survival rate, tacrolimus has several side effects that can have a severe impact on patient and graft outcomes. Since its approval, the optimal drug exposure of tacrolimus has been studied and investigated to obtain the best balance of immunosuppression in order to minimize its toxicity [82].

On the other hand, immunosuppressive therapies based on calcineurin inhibitors reduce the rate of acute cellular rejection, but can induce nephrotoxicity, neurotoxicity, metabolic disorders, and electrolyte disturbances [83,84]. A large study of deceased-donor kidney transplant patients found that five-year graft survival was equal in patients treated with cyclosporin A, tacrolimus, and mycophenolic acid or azathioprine [85]. Tacrolimus is associated with a higher risk of diabetes after KTx than cyclosporin A is, but no differences in graft or patient survival have been observed. Uncontrolled immunosuppressive therapy can increase the risk of BK virus infection and kidney impairment, and azathioprine can increase the risk of disseminated varicella zoster infection [86].

## 7. Conclusions

Graft rejection and graft loss after KTx depend on multiple factors. These risk factors can be categorized into donor-related, recipient-related, donor-recipient compatibility, and peri- and post-operative factors. Female gender, early and advanced ages, deceased donors, and concomitant diseases such as hypertension and diabetes mellitus are the main donor-related risk factors for graft rejection and graft loss. In addition, prolonged CIT might be associated with a higher risk of ischemia/perfusion injury that influences long-term graft function and survival. African American KTx recipients are vulnerable to acute rejection and graft loss. Furthermore, old age, obesity, underlying disease, prolonged dialysis, and re-transplantation are the main recipient-related risk factors that increase the probability of graft loss after KTx. Identifying these risk factors helps clinicians to avoid sub-optimal organ allocation and improves the short- and long-term outcomes of KTx. Development of new biomarkers, meticulous surgical techniques, and intensive post-transplant care, together with due attention to these risk factors, might help determine the risk of graft loss, optimize graft allocation, and improve KTx outcomes.
